# Participant Preferences for the Development of a Digitally Delivered Gardening Intervention to Improve Diet, Physical Activity, and Cardiovascular Health: Cross-sectional Study

**DOI:** 10.2196/41498

**Published:** 2023-05-02

**Authors:** Susan Veldheer, Maxfield Whitehead-Zimmers, Candace Bordner, Benjamin Watt, David E Conroy, Kathryn H Schmitz, Christopher Sciamanna

**Affiliations:** 1 Department of Family and Community Medicine Penn State College of Medicine Hershey, PA United States; 2 Department of Public Health Sciences Penn State College of Medicine Hershey, PA United States; 3 Penn State College of Medicine Hershey, PA United States; 4 Department of Kinesiology Penn State University State College, PA United States; 5 School of Medicine University of Pittsburgh Pittsburgh, PA United States; 6 Department of Medicine Penn State College of Medicine Hershey, PA United States

**Keywords:** intervention development, cardiovascular health, gardening, diet, physical activity, behavior change wheel

## Abstract

**Background:**

Low dietary intake of fruits and vegetables and physical inactivity are 2 modifiable risk factors for cardiovascular disease. Fruit and vegetable gardening can provide access to fresh produce, and many gardening activities are considered moderate physical activity. This makes gardening interventions a potential strategy for cardiovascular disease risk reduction. Previously developed gardening interventions have relied on in-person delivery models, which limit scalability and reach.

**Objective:**

The purpose of this study was to ascertain participant insight on intervention components and topics of interest to inform a digitally delivered, gardening-focused, multiple health behavior change intervention.

**Methods:**

A web-based survey was delivered via Amazon Mechanical Turk (MTurk), including quantitative and open-ended questions. Eligible participants were aged ≥20 years, could read and write in English, were US residents, and had at least a 98% MTurk task approval rating. A multilevel screening process was used to identify and exclude respondents with response inattention, poor language fluency, or suspected automated web robots (bots). Participants were asked about their interest in gardening programming, their preferences for intervention delivery modalities (1-hour expert lectures, a series of brief <5-minute videos, or in-person meetings), and what information is needed to teach new gardeners. Comparisons were made between never gardeners (NG) and ever gardeners (EG) in order to examine differences in perceptions based on prior experience. Quantitative data were summarized, and differences between groups were tested using chi-square tests. Qualitative data were coded and organized into intervention functions based on the Behavior Change Wheel.

**Results:**

A total of 465 participants were included (n=212, 45.6% NG and n=253, 54.4% EG). There was a high level of program interest overall (n=355, 76.3%), though interest was higher in EG (142/212, 67% NG; 213/253, 84.2% EG; *P*<.001). The majority of participants (n=282, 60.7%) preferred a series of brief <5-minute videos (136/212, 64.2% NG; 146/253, 57.7% EG; *P*=.16) over 1-hour lectures (29/212, 13.7% NG; 50/253, 19.8% EG; *P*=.08) or in-person delivery modes (47/212, 22.2% NG; 57/253, 22.5% EG; *P*=.93). Intervention functions identified were education and training (performing fundamental gardening and cooking activities), environmental restructuring (eg, social support), enablement (provision of tools or seeds), persuasion (offering encouragement and highlighting the benefits of gardening), and modeling (using content experts and participant testimonials). Content areas identified included the full lifecycle of gardening activities, from the fundamentals of preparing a garden site, planting and maintenance to harvesting and cooking.

**Conclusions:**

In a sample of potential web-based learners, participants were interested in a digitally delivered gardening program. They preferred brief videos for content delivery and suggested content topics that encompassed how to garden from planting to harvesting and cooking. The next step in this line of work is to identify target behavior change techniques and pilot test the intervention to assess participant acceptability and preliminary efficacy.

## Introduction

Unhealthy lifestyle behaviors such as low fruit and vegetable intake and low levels of physical activity are associated with cardiovascular disease (CVD), the leading cause of premature death in the United States [1-5]. The American Heart Association, the American College of Cardiology, and the US Preventive Task Force recommend that diet and physical activity be used for the primary prevention and treatment of CVD [6,7]. This is because of the well-established and compelling evidence demonstrating that these health behaviors can reduce the risk of premature mortality by 12%-23% [8,9]. However, despite the well-known benefits of diet and physical activity, fewer than 13% of Americans meet the minimum daily requirements of 5 servings of fruits and vegetables per day [10] and fewer than 50% of US adults meet the guidelines of 150 minutes per week of moderate or greater intensity physical activity [11]. Given the low number of Americans who meet these recommendations, new and innovative interventions are needed, which concurrently improve diet and physical activity.

Food gardening (herein referred to as gardening) has drawn the attention of public health advocates given the many investigations that demonstrate its general health benefits [12,13]. This includes positive outcomes for CVD risk factors such as BMI [14,15] and diabetes [16], in addition to factors such as mental health [17], cancer survivorship [18,19], and quality of life [20]. These studies suggest that gardening can simultaneously influence multiple health behaviors since gardeners consume more fruits and vegetables and engage in higher levels of physical activity than nongardeners—2 behavioral mechanisms that contribute to better CVD outcomes. Taken together, these findings suggest that teaching gardening skills to novice gardeners has promise as a multiple health behavior change (MHBC) intervention for CVD risk reduction [21,22].

The vast majority of published studies that teach gardening skills to adults have relied on synchronous, in-person delivery models [12,19,23-25]. In-person intervention delivery methods have inherent participation barriers due to logistical considerations such as labor availability, scheduling, transportation, and participant child care needs [26]. Over the past decade, digital interventions (eHealth) have emerged as a model with the potential to overcome some of these barriers by delivering interventions through a variety of modalities, including synchronous or asynchronous videoconferencing, email, text messages, web-based videos, and social media [26-28]. In addition, digitally delivered interventions can be cost-effective and scalable at the population level since, once developed, they can be delivered efficiently to large numbers of people [26,28-30].

While there are many potential benefits for gardening and using digital intervention delivery modes, there is a need in the larger body of behavioral eHealth literature to document the intervention development process and to integrate behavioral theory [31,32]. Such efforts allow for the potential to achieve better outcomes and improve reproducibility. One useful framework for this process is the ORBIT (Obesity-Related Behavioral Intervention Trials) model. This 4-phase model includes pre-efficacy design (phases Ia and Ib) and preliminary testing of a behavioral intervention (phases IIa and IIb), with the goal of pushing toward efficacy (phase III) and effectiveness testing (phase IV) [33,34]. In the design phase, both quantitative and qualitative methods are used in small, nonrepresentative samples to gather input from intended users on potential intervention components or strategies that may affect behavior change [33,34].

In this study, we begin at phase Ia of the ORBIT model and solicit input from a pool of potential web-based learners to gain insight on perceived needs and candidate intervention components. We included both never gardeners (NG) and ever gardeners (EG) to identify how perceptions of needs may differ based on prior experience. We used quantitative and open-ended survey questions to ascertain participant preferences for intervention delivery, topics of interest, and potential information resources that they would like to see included in a future intervention. Next, we categorized the participant comments using the COM-B (capability, opportunity, motivation, behavior) framework and intervention functions from the Behavior Change Wheel (eg, education, training, enablement) [32]. The results of this study will be used to choose potential intervention components, identify appropriate behavioral strategies, and facilitate conceptual alignment with outcome measures in an evidence-driven approach to the development of a digitally delivered MHBC gardening intervention [32].

## Methods

### Overview

Participants were Amazon Mechanical Turk (MTurk) workers who were recruited to complete human intelligence tasks (HITs) through the MTurk site in 7 batches between November 2020 and January 2021. Participants were required to be aged 20 years or older, read and write in English as their primary language, live in the United States, and have at least a 98% task approval rating from completing previous tasks. If eligible, participants were provided the survey link and were required to reach the end of the survey in order to receive a completion code. The code was the product of their Research Electronic Data Capture (REDCap; Vanderbilt University)–generated participant ID multiplied by a random number (ie, 117) and was thus unique to each participant. In addition, precautions were taken to ensure that the same worker did not take the survey multiple times, whereby workers were marked with a custom qualification through the Amazon platform, which then restricted potential workers in future batches to only those who had not previously completed the survey. Once the survey was completed, participants were further screened through a 2-level process for response inattention, thoroughness, English language fluency, and automated web robot (bot) responses [35,36].

### Screening Process

#### First-Level Screening

Prior to approving the HIT, survey responses underwent a first-level review and were only approved for compensation if (1) the worker provided the study team with a valid completion code that was associated with the correct participant ID, (2) the participant provided at least one coherent response to the open-ended questions (eg, responses were not nonsensical, were broadly relevant to the question, and words were in the appropriate order), and (3) the open-ended responses were not identical to a sentence published on the internet that was identified through a Google search (indicating a bot response).

#### Second Level Screening

Once the HITs were approved, a second-level screening occurred, which included checking for consistent responses using the following criteria: (1) age and gender demographic information was complete, and (2) open-ended responses to gardening-related questions were rereviewed based on criteria 2 and 3 from the first-level screening.

### Participant Grouping, Sociodemographic, and Health-Related Measures

Participants were grouped according to self-reported experience with gardening based on the following question: “Have you ever participated in vegetable gardening in your lifetime? Yes or no.” Those who responded “no” were labeled as NGs, and all others were considered EGs. Participants completed basic demographic questions including age, race (self-selected), Hispanic or Latino ethnicity (yes or no), sex (self-selected, male or female), and education level. Participants were asked if they had received Supplemental Nutrition Assistance in the past year (yes or no) and answered the 6-item US Department of Agriculture (USDA) food security screening questionnaire [37]. Food security responses were scored according to USDA guidelines and dichotomized as low or very low security (score: 2-6) versus food security (score: 0-1) [37]. The neighborhood of residence had self-reported options for urban, suburban, and rural.

Health-related characteristics were reported using questions from the Behavioral Risk Factor Surveillance System Survey (BRFSS) [38]. General health was assessed with a single item from the BRFSS (“Would you say that in general your health is…”), and responses were dichotomized as good or better (excellent, very good, or good) versus all others (fair or poor) [38]. BMI was calculated with self-reported height and weight (weight in kg divided by the square of the height in meters) and dichotomized using standard cut points for normal weight (BMI 18.5-24.9 kg/m^2^) versus overweight (BMI≥25). Current smokers reported smoking at least 100 cigarettes in their lifetime and currently smoking every day or some days. Participants were identified as having any CVD risk factors (yes or no) if they reported being a current smoker, had a BMI≥25, or responded positively to any of the following individual BRFSS questions: “Has a doctor, nurse or other health professional ever told you that you had any of the following? [CVD or Angina, Stroke, Heart Attack, High cholesterol, high blood pressure, Diabetes].”

### Garden Produce Perceptions and Space

We asked 6 questions about participant perceptions of homegrown fruits and vegetables and garden space availability. Perceptions of homegrown foods were assessed with 4 items: “Do you think homegrown fruits and vegetables [taste different; differ in cost; differ in quality; differ in appearance] compared to store bought fruits and vegetables? [Yes or no].” If yes, participants were further asked to choose one of 2 options: “I think homegrown fruits and vegetables [taste better or taste worse; cost less or cost more; are higher quality or are lower quality; have a better appearance or have a worse appearance] than store-bought fruits and vegetables.” Questions about garden space included a root question asking: “Where you live now, do you have any space outside where you can grow fruits, vegetables, and herbs? [yes or no]” and “Is there space for you to grow plants in pots? [yes or no]” with responses to describe this outdoor space as “in pots on a patio or deck, in pots on a balcony, or in pots on the ground.” Responses were categorized as no outside space versus any.

### Cooking Skills

Three cooking-related constructs were assessed, including food agency, cooking skills, and food skills [39,40]. These constructs are associated with higher consumption of fruits and vegetables in adults [41] and were included because they are a natural prerequisite for consuming homegrown produce. Food agency was assessed using the Cooking and Food Provisioning Action Scale and scored using recommended analytical methods, yielding a total score and 3 subscale scores: cooking self-efficacy, attitudes, and structural barriers [40]. Cooking skills, related to cooking methods and preparation techniques, were assessed with 14 questions on a 7-point scale (very poor=1 to very good=7) and a total score was calculated by summing the points from each question (score range 14-98). Similarly, food skills related to meal planning, shopping, budgeting, and cooking resourcefulness were assessed with 19 questions on a 7-point scale (very poor=1 to very good=7), and a total score was calculated by summing the points from each question (score range 19-133) [39].

### Gardening Program Interest and Delivery Format

To assess interest in participating in a gardening program, participants were asked the following question using a 3-option choice format used in previous clinical studies to gauge interest in future programs [42,43]: “Would you consider participating in any of the following programs if offered for free? (1) A series of 1-hour web-based sessions offered weekly where experts would lecture on a variety of gardening topics (yes or no), (2) a series of brief YouTube videos (less than 5 minutes) that provide “how-to” instructions on specific gardening topics (yes or no), and (3) a community gardening program where you and other people would meet in-person a few times a month to learn how to garden (yes or no).” Options 1 and 3 were chosen based on the educational formats commonly offered through national cooperative extension programs [44,45], and option 2 was based on formats used in popular social media video platforms (eg, YouTube and TikTok). An affirmative response to any of these 3 options was coded as “any interest” in a gardening program. For delivery method preference, participants were also asked, “Of these programs, which do you think is the most interesting?” and could choose one of the 3 options.

### Qualitative, Open-ended Questions

Participants were asked to complete 3 open-ended questions to understand what information they would consider important in a gardening program, including: (1) “If we were going to create a gardening program, what kinds of things would you like to see included?”; (2) “What information do you think you (or someone who is new to gardening) would need to start a vegetable garden on your (their) own?”; and (3) “What types of information sources would you most want [were helpful for you] to learn about how to start a vegetable garden?” One additional question also asked what sources of information participants would use to learn about gardening.

### Qualitative and Quantitative Analytical Methods

We began the analysis of the qualitative open-ended questions with 2 investigators (SV and MWZ) independently reviewing all the participant responses. Using the constant comparative method, we identified words or phrases with like meaning and unitized these phrases with codes [46,47]. We then met to establish a formal codebook with illustrative participant phrases for each code. Disagreements were discussed, and the codebook was modified as needed. Both investigators coded an initial sample of the data and achieved a high level of interrater reliability (Cohen κ=.82) [48]. Once all the data were coded, we organized the codes into intervention function categories based on Michie et al’s Behavior Change Wheel [32]. To assist with visualizing the relative importance of each intervention function, we presented counts of how many times each intervention function was mentioned and then calculated the percent of the total comments. Comments related to intervention delivery modalities (eg, “provide videos”) rather than intervention function were removed from the intervention function counts but were retained, coded, and reported separately. The quantitative data were summarized, and differences between NG and EG were assessed using *t* tests and chi-square tests, as appropriate.

### Ethics Approval

This study was conducted according to the guidelines of the Declaration of Helsinki and approved by the Penn State University Institutional Review Board (protocol #15352, approved June 14, 2020). All participants were provided a written summary explanation of the research and completed informed consent prior to providing study data. All study data were collected using REDCap [49] to ensure participant privacy and confidentiality. All study data were collected anonymously, and participants are not individually identifiable. Participants were compensated US $3 via the Amazon MTurk platform.

## Results

A total of 808 MTurk workers started the survey and provided a completion code. Of them, 91 were excluded based on the first level screening review. During the second level review, an additional 250 were excluded owing to a combination of missing demographics (n=244) and inconsistent responses (n=8), leaving an analytic sample of 465 participants (212 NG and 253 EG). Participants resided in 43 states within the continental US. The sociodemographic characteristics, health-related outcomes, perceptions of home-grown fruits and vegetables, and cooking skills of the sample overall and by group are presented in Table 1. EGs reported participating in gardening for an average of 7.3 (SD 9.1) years. There were few sociodemographic differences between NG and EG with the exception that EG were significantly older and somewhat less likely to live in urban areas than NG. Similarly, the only difference in health-related characteristics is that the EG group had a lower BMI than NGs (*P*=.045). The majority of participants believed that home-grown fruits and vegetables (compared to store bought) tasted better, cost less and were higher quality and the proportion of participants who endorsed this belief was highest in the EG group. Most participants also believed that store-bought fruits and vegetables had a better appearance than home-grown. Food agency, cooking skills, and food skills were significantly lower for the NG than EG in all domains.

Overall, 72.3% (n=336/465) of participants had outdoor space available for gardening (either in containers or in the ground) although this varied by gardening group (NG: 120/212, 56.6%; EG: 216/253, 85.4%; *P*<.001). Of those who had outdoor space (NG, n=120; EG, n=216), 18.8% (63/336) had space only for container gardening which also differed by group (NG: 36/120, 30%; EG: 27/216, 12.5%; *P*<.001). There was a high level of program interest overall (355/465, 76.3%), though interest was higher in EG (142/212, 67% NG; 213/253, 84.2% EG; *P*<.001). There were no differences between the groups for the program delivery mode and the majority of participants (282/465, 60.7%) preferred a series of brief <5-minute videos (136/212, 64.2% NG; 146/253, 57.7% EG; *P*=.16) over 1-hour lectures (29/212, 13.7% NG; 50/253, 19.8% EG; *P*=.08) or in-person delivery modes (47/212, 22.2% NG; 57/253, 22.5% EG; *P*=.93).

**Table 1:** Sociodemographic and health-related characteristics of study participants overall and comparisons between Never Gardeners (NG) and Ever Gardeners (EG).^a^

Characteristic or measure				Overall (N=465)		NG (N=212)		EG (N=253)	*P* value^b^
**Sociodemographic characteristics**									
	Age (years), mean (SD)		40.2 (11.8)		36.9 (9.8)		43.0 (12.7)		<.001
	Female, n (%)		219 (47.2)		92 (43.4)		127 (50.2)		.14
	White, n (%)		371 (79.8)		161 (75.9)		210 (83.0)		.06
	Hispanic or Latino ethnicity, n (%)		38 (8.2)		22 (10.4)		16 (6.3)		.11
	Married or partnered, n (%)		316 (68.0)		144 (67.9)		172 (68.0)		.99
	Bachelor’s degree or higher, n (%)		315 (67.7)		148 (69.8)		167 (66.0)		.38
	Low or very low food security, n (%)		205 (43.9)		102 (48.1)		101 (39.9)		.08
	Received SNAP^c^ (past year), n (%)		151 (32.5)		64 (30.2)		87 (34.4)		.34
	**Self-reported neighborhood, n (%)**								.001
		Urban	201 (43.2)		100 (47.2)		101 (39.9)		
		Suburban	194 (41.7)		94 (44.3)		100 (39.5)		
		Rural	70 (15.1)		18 (8.5)		52 (20.1)		
**Health-related characteristics,** **n (%)**									
	In good health or better		428 (92.0)		196 (92.5)		232 (91.7)		.77
	BMI≥25 (kg/m^2^)		207 (48.6)		107 (53.8)		100 (44.1)		.045
	Current smoker		101 (21.7)		42 (19.8)		59 (23.3)		.36
	Prediabetes or diabetes		45 (9.7)		16 (7.6)		29 (11.5)		.16
	High cholesterol		62 (13.3)		26 (12.3)		36 (14.2)		.54
	History of coronary artery disease		16 (3.4)		6 (2.8)		10 (4.0)		.51
	History of heart attack or angina		14 (3.0)		6 (2.8)		8 (3.2)		.84
	History of stroke		19 (4.1)		9 (4.3)		10 (4.0)		.87
	History of deep vein thrombosis		20 (4.3)		9 (4.3)		11 (4.4)		.96
	Family history of early CVD^d^		78 (16.8)		29 (13.7)		49 (19.4)		.10
	With at least 1 risk factor for CVD		342 (73.6)		156 (73.6)		186 (73.5)		.99
**Perceptions of home-grown F&V^e^ compared to store-bought ones, n (%)**									
	Home-grown taste better		340 (72.8)		123 (58.2)		215 (85.0)		<.001
	Home-grown cost less		300 (64.2)		118 (55.7)		182 (71.9)		.001
	Home-grown F&V are higher quality		324 (69.4)		117 (55.2)		205 (81.0)		<.001
	Home-grown F&V have a better appearance		188 (40.3)		63 (29.7)		123 (48.6)		<.001
**Cooking skills, mean (SD)**									
	**Mean total cooking agency score**		12.2 (2.4)		11.7 (2.3)		12.7 (2.5)		<.001
		Cooking attitudes	4.0 (1.0)		3.8 (0.96)		4.2 (1.00)		<.001
		Structural skills	3.1 (1.0)		2.9 (0.92)		3.1 (1.01)		.02
		Functional skills	5.1 (1.0)		4.8 (1.06)		5.3 (0.89)		<.001
	Mean cooking skills score		73.7 (15.6)		68.8 (17.1)		77.8 (13.0)		<.001
	Mean food skills score		102 (18.7)		96.4 (19.9)		106.7 (16.1)		<.001

^a^NG reported 0 years of gardening experience, EG reported 1 or more years of gardening experience.

^b^Chi-square tests and independent *t* tests were used to compare differences between the groups.

^c^SNAP: supplemental nutrition assistance program.

^d^CVD: cardiovascular disease.

^e^F&V: fruits and vegetables.

The frequency and ranking of the participants' comments from the qualitative, open-ended questions, organized by intervention function and group are presented in Table 2. A total of 871 comments were received from 398 participants (85.6% of overall sample). The most commonly suggested intervention functions for both groups were education and training. Environmental restructuring (eg, social support) was the next most commonly suggested for NG although this was rated much lower by EG. A total of 132 comments were related to intervention delivery rather than function. Generally, participants suggested that the intervention should be “short and simple” and oriented “for beginners,” in addition to including “tips and tricks,” videos, “hands-on” activities, and books. Both groups suggested that they would use similar sources of information to learn about gardening with the internet or social media being the most common option followed by experienced gardeners or friends, and books.

**Table 2:** Frequency of participant comments ranked by the percentage of total comments for Never Gardeners (NG) and Ever Gardeners (EG) organized by the capability, opportunity, motivation, and behavior (COM-B) model, and linked to the Behavior Change Wheel intervention functions from Michie et al [32].

Participant response summary and illustrative comments		NG,^a^ Rank	NG, n (%)	EG,^a^ Rank	EG, n (%)
**Capability: Education and Training^b^**					
	**The garden maintenance basics**: “Start with very basic things like mulching, weeding, manuring or fertilizing, and watering.”	1	85 (26.0)	1	93 (18.1)
	**How and when to start seeds, plant, and harvest:** “I would like to see how to plant common veggies and when to plant them and when to harvest them.”	2	55 (16.8)	2	91 (17.7)
	**Preparing the soil and fertilizer:** “Information on the best vegetables to use with the soil in my location”; “Using the right fertilizer.”	3	35 (10.7)	3	82 (15.9)
	**Choosing seeds/plants for your location:** “How to pick appropriate plants for your area”	4	28 (8.6)	4	51 (9.9)
	**Specialty gardening (indoor gardens, small spaces):** “Small space gardening, especially with pots”; “I'd like to see indoor gardening for small spaces”	5	21 (6.4)	7	31 (6.0)
	**Tools and supplies:“**What tools and supplies you need”; “I would like to see how to get started with basic tools.”	7	18 (5.5)	12	10 (1.9)
	**How to use produce/cooking: “**Tips on how to cook certain veggies, easy meal plans, easy recipes that are kid friendly”; “What parts of the plant are edible.”	8	16 (4.9)	6	35 (6.8)
	**Pest control (including disease, insects, animals):** “…Keeping animals out of the plants”; “How to control pests organically.”	9	13 (4.0)	5	44 (8.5)
	**Money saving/tips for high yields:“**Which vegetables or fruit are easiest and most cost saving”; “Low cost and effective ways to garden with easy to follow instructions.”	11	9 (2.8)	10	15 (2.9)
**Opportunity: Enablement**					
	**Provide gardening items (seeds, space, supplies):** “I would like to see gardening equipment provided and a variety of different vegetables to grow.”	14	3 (1.0)	14	0 (0)
**Opportunity: Environmental Restructuring (Social)**					
	**Facilitate group interaction and activities:** “Families being welcomed and encouraged to join in”; “Group projects”; “Open discussions with the community members”; “Discussion forums for help”	6	19 (5.8)	9	21 (4.1)
**Motivation: Persuasion**					
	**Offer encouragement and highlight the benefits of gardening (including personal satisfaction):** “Have patience”; “Benefits of having a garden”; “How nice it is when [it is] all grown and you get to pick the vegetables that you have put all the work into.”	10	12 (3.7)	8	25 (4.9)
**Motivation: Modeling**					
	**Use content experts:** “Use experts in your program”; “Nice professionals who know about gardening.”	12	9 (2.8)	11	13 (2.5)
	**Testimonials or garden tours:** “People that actually did it from home and show results”; “We can show off our progress on our own gardens”; “Before and after photos”; “show and tell”	13	4 (1.2)	13	4 (0.8)

**^a^**A total 398 participants who provided 871 comments (346 NG and 525 EG).

**^b^**Education and training have distinct functions but they are presented together because participant comments were unclear as to which category they were referring.

## Discussion

### Main Findings

This ORBIT phase Ia study solicited input from potential web-based learners to inform the development of a future digitally delivered, MHBC gardening intervention. The majority of participants expressed interest in the intervention topic area and indicated that a web-based format using a series of brief videos would be preferable over webinar-based lectures or in-person models. Participants also suggested the inclusion of 6 different intervention functions based on the COM-B framework from Michie et al [32] including education, training, environmental restructuring (eg, social support), persuasion, enablement, and modeling. Below we will consider the results of this study through the lens of COM-B to examine the specifics of how the identified intervention functions may be linked to this model in the context of a gardening intervention.

Capability involves an individual’s knowledge or skills and can be described as the psychological or physical capacity to engage in a particular behavior [32]. Capability is linked to 2 related, but distinct COM-B intervention functions including education (imparting knowledge) and training (achieving skills). It is also in alignment with social cognitive theory which posits that knowledge is an important element for behavior change [50]. Both NG and EG were fairly consistent in suggesting the need for gardening-related knowledge and skills and the topics they identified were broadly consistent with other programs that have offered gardening-based education [18,23,44,45].

The participants identified cooking knowledge and skills as areas of interest and this is encouraging since preparing garden produce is a necessary prerequisite to consuming it. Other investigators have found that both gardening and cooking are independently and positively associated with higher-quality diets [41,51]. In our sample, EG had significantly higher scores than NG on all included measures of cooking agency. To our knowledge, this is the first study to measure cooking skills in a sample of gardeners and nongardeners and it raises the question: “Are gardeners more likely to have strong cooking skills or are people with strong cooking skills more likely to garden?” It seems entirely possible that this relationship is bidirectional. Perhaps, among gardeners, the arrival of fresh garden produce encourages them to cook more leading to better cooking skills. Conversely, perhaps skilled cooks more highly value fresh fruits and vegetables and begin gardening as a way to procure high-quality produce. Either way, our data suggest a potential relationship between gardening and cooking that warrants further exploration in future work.

Opportunity in the COM-B model includes the factors that lie outside of the individual and can encompass both physical and social aspects [32]. In this study, a surprisingly low number of participants (3 NG and 0 EG) suggested that physical enablement, or the need for space, tools, seeds, and other supplies, be included in an intervention. It is possible that both NG and EG already have what they need to engage in gardening. However, for NG, it is equally plausible that they are unaware of the materials needed to engage in gardening. Given that the provision of gardening tools and supplies would add to the cost of delivering an intervention, further exploration is warranted to understand what participants may need to enable gardening activity. In addition, while gardening materials can be supplied in the context of a gardening intervention, helping participants identify outdoor space to garden either in the ground, in containers, or a community garden setting is a critical barrier to overcome.

When considering space limitations, it is tempting to assume that gardeners are less likely live in urban settings. However, in a representative sample of US adults, Kegler et al [15] found that 30% of US adults reported gardening and while the proportion of gardeners was lower in urban settings (28.5%), the difference was not as striking as might be expected (32.9% semiurban, 35.9% rural). One reason that gardening is feasible in urban settings is that a meaningful amount of produce can be grown in a relatively small space. Conk et al [52] found that in a small garden bed (4'×8'), a gardener can produce up to 320 servings of vegetables over a single growing season. This is sufficient to meet 100% of that individual’s USDA-recommended vegetable servings for 4 months [52]. Importantly, while the majority of gardening that occurs in the US is home-based (92%), there are many practical options for outdoor gardening in small spaces and urban settings. This includes growing in pots or containers on apartment balconies, in small front or back yards [53], or by accessing community gardens which are located in all 50 states [54,55], at schools [56], and on medical center campuses [23,57,58].

In this study, both NG and EG identified small-space gardening as a topic of interest. However, it was not clear from their responses how small these spaces might be (ie, a pot of thyme on a windowsill or a network of pots on a balcony). Given that our interest is in the potential for gardening to improve CVD outcomes, future work is needed to understand these comments and to examine if small-space gardening can provide meaningful amounts of fruits and vegetables or physical activity.

Finally, motivation in the COM-B model includes internal processes that energize and direct behavior [32]. This is in alignment with self-determination theory, which posits that activities that garner enjoyment, personal accomplishment, and excitement (eg, internal motivation), are better able to be sustained over long periods of time [59]. A unique aspect of gardening is that although it is both a means to increase household fruit and vegetable availability and physical activity, these are not typically the reasons that gardeners cite for engaging in it. Chalmin-Pui et al [60] surveyed gardeners in the United Kingdom (n=5776), to understand gardeners’ motivations and while participants did refer to the health benefits of gardening, there were many other motivations identified including pleasure, seeing plants grow, an expression of self-identity, relaxation, and environmental sustainability.

In this study, EG commented on the personal satisfaction that arises from gardening by stating “how nice it is” when you “get to pick the vegetables.” They also valued garden produce and were significantly more likely to rate garden produce higher than store-bought produce for characteristics such as cost, flavor, and quality. Taken together, these findings suggest that a future gardening intervention could be positioned to appeal to non-health–related values; an approach that has been called a stealth intervention [61,62]. Stealth interventions are designed to engage people in a target activity (eg, gardening) that indirectly promotes the desired behavior (eg, physical activity and improved diet) by leveraging peoples’ interests and values (eg, pleasure, enjoyment, agency, cost-effectiveness, and sustainability). It seems plausible that a future gardening intervention could achieve positive health outcomes by explicitly targeting internal motivational aspects of enjoyment and personal satisfaction rather than health benefits.

After education and training, the next largest proportion of participant suggestions was related to environmental restructuring of the social environment including social support, group interaction, discussions, and feedback. These comments raise the question as to whether a digitally delivered intervention needs to include in-person components or whether digital person-to-person interactions are adequate. Santarossa et al [63] take up this question in a recent narrative review and suggest that investigators should take a new “Web 2.0” perspective to eHealth intervention development. This perspective considers not just asynchronous delivery models but also person-to-person synchronous intervention components [63]. The authors suggest that high-quality, in-person connections can be facilitated through digital person-to-person components (eg, videoconferencing) and that these interactions are likely to be as effective as in-person social support. Though published prior to the COVID-19 pandemic, the Santarossa et al [63] commentary provides a timely option for integrating person-to-person interactions into a post-pandemic intervention.

### Limitations

This study has limitations and strengths. Previous investigators have noted some concerns about the use of the MTurk platform for survey distribution [64]. In the context of this study, relevant concerns are focused on three main issues including (1) the representativeness of the MTurk worker pool, (2) issues of attentiveness, and (3) the presence of bots. Consistent with ORBIT model phase 1a, this study was not designed to be representative but rather our goal was to solicit input that can provide direction for the development of future interventions. Thus, the results of this study provide useful insights that can be used for intervention development in addition to generating new ideas that can be tested in future studies. A significant strength of our study is that we used previously published strategies to ensure high-quality MTurk samples including the use of written open-ended comments to screen for English language fluency, screening for bot responses taken from other locations on the internet, and removing participants with nonsensical responses [35,36,64]. This, in addition to the inclusion criteria we used (eg, 98% HIT approval rating, requiring a unique completion code, and limiting duplicate responses with the use of the custom qualification through the Amazon platform), provides reassurance that responses from our participants are valid and can be used to draw the conclusions presented in this report.

Finally, our results are based on the acceptability of a hypothetical intervention. It is possible that what participants want in an intervention may not be practically feasible to deliver, may not fit what is known by behavioral scientists to optimize behavior change, or may not confer cardiovascular benefits (ie, small space gardening). In addition, gardening is complex and relies not only on individual behavior change, but is also dependent on the external environment (eg, soil conditions, weather, and pests) that may be outside the control of an individual or what an intervention can address. Future work is needed to develop an intervention that balances what can practically be delivered with fidelity in a digital environment, the recommendations made by the participants in this report, and behavior change strategies known to be effective.

### Conclusions

In this ORBIT Phase 1a study, potential web-based learners were interested in a digitally delivered gardening program, they preferred brief videos for content delivery (<5 minutes), and they suggested content topics that encompassed how to garden from planting to harvesting and cooking. Participant comments and health behavior theory support incorporating opportunities for gardening knowledge and skill development while also fostering social support and highlighting the enjoyable and personally satisfying aspects of gardening. The next step in this line of work is to identify target behavior change techniques, develop an intervention delivery strategy that engages these targets, and conduct pilot testing to assess participant acceptability and preliminary efficacy of the newly developed intervention.

## Abbreviations

BRFSS: Behavioral Risk Factor Surveillance System Survey
COM-B: capability, opportunity, motivation, behavior
CVD: cardiovascular disease
EG: ever gardeners
HIT: human intelligence tasks
MHBC: multiple health behavior change
MTurk: Mechanical Turk
NG: never gardeners
ORBIT: Obesity-Related Behavioral Intervention Trials
USDA: US Department of Agriculture

## Data Availability

The data sets generated during or analyzed during this study are available from the corresponding author upon reasonable request.
